# The Evolving Landscape in Multiple Myeloma: From Risk Stratification to T Cell-Directed Advanced Therapies

**DOI:** 10.3390/cancers17030525

**Published:** 2025-02-05

**Authors:** Carmen Besliu, Alina Daniela Tanase, Ionela Rotaru, Jose Espinoza, Laura Vidal, Martine Poelman, Manel Juan, Carlos Fernández de Larrea, Kamal S. Saini

**Affiliations:** 1 Fortrea Inc., 8 Moore Drive, Durham, NC 27709, USA; carmen.besliu@fortrea.com (C.B.); jose.espinoza@fortrea.com (J.E.); laura.vidalboixader@fortrea.com (L.V.); martine.poelman@fortrea.com (M.P.); 2 Department of Hematology and Bone Marrow Transplantation, Fundeni Clinical Institute Bucharest, 022328 Bucharest, Romania; alina.tanase@icfundeni.ro; 3 Department of Hematology, Municipal Hospital Craiova, 010024 Craiova, Romania; ionela.rotaru@umfcv.ro; 4 Institut d’Investigacions Biomèdiques August Pi i Sunyer (IDIBAPS), Hospital Clinic de Barcelona, 08036 Barcelona, Spain; mjuan@clinic.cat (M.J.); cfernan1@clinic.cat (C.F.d.L.); 5 Addenbrooke’s Hospital, Cambridge University Hospitals NHS Foundation Trust, Cambridge CB2 0QQ, UK

**Keywords:** multiple myeloma, risk stratification, immunotherapy, CAR T cell therapy, bispecific antibody, BCMA, CD38, GPRC5D

## Abstract

Multiple myeloma is biologically and clinically a complex and heterogeneous disease which develops late in life, with the median age at the time of initial diagnosis being 66 years. In 1975, Durie and Salmon developed the first broadly adopted staging system in multiple myeloma, and in the ensuing decades, the risk stratification tools have improved and now incorporate different parameters to better predict the prognosis and to guide the treatment decisions. The International Staging System (ISS) was initially developed in 2005, revised in 2015 (R-ISS), and again in 2022 (R2-ISS). Tremendous progress has been achieved in multiple myeloma therapy over the past 25 years with the approval of immunomodulatory drugs, proteasome inhibitors, and anti-CD38 monoclonal antibodies, resulting in a major paradigm shift. The dysfunction of the innate and adaptive immune system, especially in the T cell repertoire, represents a hallmark of multiple myeloma evolution over time, supporting the need for additional therapeutic approaches to activate the host’s immune system and to overcome the immunosuppressive tumor microenvironment. Novel T cell-directed therapies include chimeric antigen receptor (CAR) T cell therapies and bispecific antibodies that leverage the immune system’s T cells to recognize and attack the tumor cells. Second-generation anti-BCMA CAR T cell therapies and bispecific antibodies that bind the tumor antigen BCMA or GPRC5D onto myeloma cells and CD3 on the T cell’s surface are currently available for the treatment of relapsed/refractory multiple myeloma. Despite impressive results obtained with currently approved treatments, multiple myeloma remains incurable, and almost all patients eventually relapse. Moreover, patients with extramedullary disease and plasma cell leukemia represent an unmet medical need that require additional strategies to improve the outcome. In this review, we provide an overview of the evolution of risk stratification and the treatment of multiple myeloma.

## Simple Summary

Multiple myeloma is a plasma cell cancer accounting for 10% of all blood malignancies, and the incidence of the disease has gradually increased over time. In recent decades, great progress has been made in understanding the disease biology, leading to an improvement in outcomes. However, despite the availability of several treatment options, multiple myeloma remains essentially incurable, and most patients eventually relapse. Efforts are being made to further improve the outcome of the patients with newly diagnosed and relapsed disease by developing new agents, especially T cell-directed therapies. Researchers also continue to test different drug combinations and sequences. This review article provides an overview of recent advances in the field.

## 1. Introduction

Multiple myeloma is a plasma cell malignancy accounting for about 1% of all cancers. It represents 10% of all hematologic malignancies and is the second most common hematological neoplasm after lymphomas [1]. The global burden of the disease has increased over the years, and the International Agency for Research on Cancer (IARC) estimates a worldwide incidence of 187,952 new cases and 121,388 deaths due to multiple myeloma in 2022 [2]. A statistical modeling study conducted in Australia projected that the age-standardized incidence rate will increase by 14.9%, while the mortality rate will decline by 27.5% between 2018 and 2043, which will lead to an increased prevalence of multiple myeloma cases over the next 25 years [3]. The disease develops later in life, and at the time of diagnosis, the median age is 66 years, with approximately 38% aged 70 or older and only 2% of patients being younger than 40 years [4].

Multiple myeloma is biologically and clinically a complex and heterogeneous disease characterized by the clonal proliferation of plasma cells in the bone marrow and the production of monoclonal protein, leading to significant morbidity mainly due to bone disease and associated features such as anemia, hypercalcemia, and an increased risk of infection and renal impairment, finally, resulting in multiple end-organ damage.

Much progress has been made over the past decades in understanding myeloma biology, especially the cytogenetic and molecular characterization of the disease, which has led to improvements in early diagnosis, risk stratification, and the management of the disease [5].

The treatment landscape has dramatically changed with the introduction of immunomodulatory drugs (IMiDs), proteasome inhibitors (PIs), and immune therapies such as anti-CD38 monoclonal antibodies (mAbs) (2015) [6] and more recently with the approval of chimeric antigen receptor (CAR) T cell therapy (2021) [7] and bispecific antibodies (2022) [8], leading to a significant improvement in patient outcomes. The advances of the past decades in patient management led to an increase in median survival of patients with multiple myeloma from about 3 years to 8–10 years [9]. Hematopoietic stem cell transplantation remains an important treatment strategy in transplant-eligible, newly diagnosed multiple myeloma (NDMM), while other new drugs such as the antibody–drug conjugate (ADC) belantamab mafodotin and the nuclear export inhibitor selinexor have added to the armamentarium against relapsed or refractory multiple myeloma (RRMM) [10].

For this manuscript, we reviewed published clinical trials results, approved labels of the drugs, professional society guidelines, and conference proceedings up to September 2024.

## 2. Risk Stratification of Multiple Myeloma over the Past Decades

The survival of patients with multiple myeloma depends on several factors including demographic features, tumor burden, the biology of the disease, and the response to the treatment [11]. Risk stratification has greatly improved over time as a result of updates to the scoring systems to incorporate different emerging parameters that better predict the prognosis and guide treatment decisions [10,12].

The first widely adopted staging system used as a prognostic tool in multiple myeloma was developed in 1975 by Durie and Salmon and included parameters that reflect the myeloma tumor burden such as hemoglobin and serum calcium levels, the number of bone lesions, and the level of monoclonal protein. The Durie and Salmon system (DSS) remained a standard staging and prognostic tool to predict tumor load and estimate survival for NDMM patients for many years [13].

In 2005, the International Staging System (ISS) was developed, and it was widely accepted as a prognostic tool, often superseding DSS. The ISS was based on easily measurable, objective, and reproducible parameters, such as serum beta-2 microglobulin (Sβ2M) and albumin levels, and provided a more equal distribution of patients among the stages [14] (Table 1). However, like DSS, the ISS did not take into account the cytogenetic features of the disease. Although it is a robust and validated system, it remains unclear whether higher ISS stages reflect higher tumor burden, the level of organ damage, or both. Moreover, in patients treated upfront with autologous stem cell transplantation (ASCT), the ISS could not improve the prediction of post-transplant outcomes compared to DSS [15], yet it remains clinically informative in the era of CAR T cell therapies.

The Mayo Clinic Stratification of Myeloma and Risk-Adapted Therapy (mSMART) classification, which was initially published in 2007 and updated in 2013 (www.msmart.org (accessed on 30 August 2024)), incorporates cytogenetics as a part of the risk stratification. However, many clinicians do not consider mSMART to be a prognostic system but rather a tool to inform and guide treatment strategies [18,19].

The revised International Staging System (R-ISS) proposed by International Myeloma Working Group (IMWG) in 2015 combines ISS with cytogenetic abnormalities and lactate dehydrogenase (LDH) levels to create a unified prognostic index that incorporates both tumor burden and the biological features of the disease, including three high-risk cytogenetic markers: del(17p), translocation t(4;14), and t(14;16) [16] (Table 1).

In 2022, the European Myeloma Network (EMN), as part of the Harmony project, proposed the second revision of the ISS (R2-ISS), incorporating the newly identified poor prognostic factor chromosome 1q+ gain/amplification (present in approximately 40% of NDMM), allowing for a better stratification of the intermediate-risk NDMM patient group and representing an important step forward [17].

A validation of R2-ISS was conducted by the Balkan Myeloma Study Group (BMSG) in a real-world myeloma cohort in 2023. The study included 1503 patients with a median age of 66 years, and the 5-year overall survival (OS) rate for the four R2-ISS stages was 76%, 70%, 48%, and 38% (*p* < 0.001), with no significant differences between stage I and II groups but significant differences for stage II vs. III and stage III vs. IV (*p* < 0.001). Stage I and II groups also had similar outcomes in transplanted vs. non-transplanted patients. The data indicate that R2-ISS discriminates patients at high risk with more accuracy [20].

Maura et al. recently published the results of the GMMG-HD6 clinical trial which evaluated the use of the prognostic model to predict the individualized risk in NDMM (IRMMa) based on 20 genomic features. The authors concluded that the model was able to better identify patients at risk of primary refractory and early progressive myeloma and those patients for whom ASCT was more effective. In addition, the accuracy of overall survival (OS) was significantly higher than that obtained using previous prognostic models (ISS, R-ISS, and R2-ISS) [21].

Other clinical factors such the level of circulating plasma cells, plasma cell leukemia, extramedullary disease (EMD), and renal failure have poor prognostic implications [22]. The reported incidence of EMD varies considerably, mainly due to the variation in definition, and is noted in up to 5% of patients during the initial diagnostic workup and in up to 20% of RRMM. EMD implies an inferior prognostic outcome even in the era of CAR T cell therapies, emphasizing the need for new treatment strategies to enhance patients’ outcome [23].

Physical frailty rather than chronologic age or the performance status of patients has been recognized as an independent predictor factor for survival and risk for toxicity in elderly myeloma patients. In an analysis conducted by Palumbo et al. in 869 patients with NDMM with a median age of 75 years where 46% of patients were older than 75 years, the 3-year OS was 84% in fit, 76% in intermediate-fitness, and 57% in frail patients; the cumulative incidence of grade ≥ 3 non-hematological toxicities and treatment discontinuations were higher in frail patients compared to fit or intermediate-fitness groups. A frailty scoring system based on patient age, comorbidities, and functional status was proposed by IMWG [24]. In addition, in a Myeloma XI trial, the progression-free survival (PFS) and OS displayed worsening clinical outcomes with each decade increase in the age of the patient. The frequency of t(4;14) and del(17p) decreases, and gain(1q) increases with age, postulating that the impact of the genetic factors decreases with age while frailty can be an important predictive factor for outcome in the elderly population which needs to be incorporated while stratifying the patients to deliver personalized treatment approaches [25].

The risk of multiple myeloma recurrence is dynamic and is impacted by the degree of response to the initial treatment, particularly the presence and magnitude of minimal residual disease (MRD). With recent therapeutic advances in the last few years, including quadruplet combinations, CAR T cell therapy, and bispecific antibodies, a substantial number of patients with NDMM and RRMM are now expected to achieve complete response (CR) or better. The ≥CR rate was 87.9% in transplant-eligible NDMM with the Dara-VRd regimen in the PERSEUS trial [26] and 74.7% in transplant-ineligible NDMM with Isa-VRd in the IMROZ trial [27], while in patients with lenalidomide-refractory disease, the ≥CR rate was 73.1% with CAR T cell therapy ciltacabtagene autoleucel (Cilta-cel) in the CARTITUDE-4 trial [28] and 39.4% in patients with RRMM and multiple prior lines of therapy treated with the T cell engager bispecific antibody teclistamab in the MajesTEC-1 trial [29].

Although CR is strongly associated with longer PFS and OS [30], many patients who achieve CR still progress. This underlines the need for a new response category to identify deeper responses beyond those conventionally defined as CR/sCR. In 2016, the IMWG recommended an MRD assessment at CR/sCR, with a minimum sensitivity of one nucleated tumor cell in 100,000 normal cells (10^−5^ sensitivity threshold), via either next-generation sequencing (NGS) or next-generation flow cytometry [31]. The association between the depth of response/MRD and long-term outcome was evaluated in several pooled analyses. A large meta-analysis of 44 studies with data for PFS and 23 studies with data for OS reported improved PFS (HR, 0.33; 95% CI, 0.29–0.37; *p* < 0.001) and OS (HR, 0.45; 95% CI, 0.39–0.51; *p* < 0.001) in patients achieving negative MRD compared to a positive MRD status, regardless of disease settings, cytogenetic risks, the method of MRD assessment, sensitivity threshold, or depth of clinical response at the time of MRD assessment [32]. Patients who achieve complete remission or better and a MRD-negative status show improved PFS compared to patients who are MRD-positive with CR or better [33].

Furthermore, in April 2024, the FDA’s Oncology Drug Advisory Committee voted in favor of using MRD as an intermediate endpoint in clinical studies to support accelerated approval for new drugs in multiple myeloma [34].

## 3. Evolution of Multiple Myeloma Therapy

The treatment of multiple myeloma has remarkably improved since 1844, where the first documented case of “mollities ossium” was described in a 39-year-old woman, Sarah Newbury, who was treated with rhubarb pills and an infusion of orange peels, and another patient, Mr. McBean, who obtained some response to steel and quinine one year later [35,36]. A century later, in 1947, urethane was introduced as a treatment for multiple myeloma after N. Alwall communicated the benefit of the drug on reducing the serum globulin and proteinuria levels and decreasing the plasma cells in bone marrow [37].

## 4. Cytotoxic Chemotherapy, Immunomodulators, and Stem Cell Transplantation

Urethane was the standard of care for multiple myeloma till the introduction of the alkylating agent melphalan, the first breakthrough treatment showing consistent responses in myeloma patients with a significant reduction in tumor burden and an improvement of symptoms. Early data on the clinical benefit in patients with myeloma treated with melphalan were reported in 1958 by Blokhin [38]. The combination of melphalan and prednisone (MP) was established as a standard regimen for multiple myeloma after R. Alexanian communicated, in 1969, the results of a randomized clinical study in which survival was improved by 6 months with the combination of MP compared with melphalan alone [39]. Several other chemotherapy combinations were tested in myeloma, including the so-called M-2 protocol. However, a meta-analysis performed in 1998 by the Myeloma Trialists’ Collaborative Group to compare combination chemotherapy (CCT) versus melphalan plus prednisone (MP) concluded that although the response rates were higher with CCT versus MP (60% vs. 53%, *p* < 0.001), there were no differences in overall survival; thus, the MP combination remained the backbone of myeloma treatment [40].

Until the mid-90s, the median survival of patients after conventional chemotherapy was about 3 years [41]. With the introduction of ASCT with high-dose chemotherapy (HDCT), the median survival improved to 7 years [42]. The first ASCT in multiple myeloma was reported by McElwain and Powles in 1983 in a patient with plasma cell leukemia after conditioning regimen with melphalan [43]. High-dose chemotherapy supported by ASCT improved the response rates and survival compared to conventional therapy alone. In a study published by Attal et al. in 1996, the estimated OS at 5 years was 52% in the ASCT group vs. 12% in the conventional chemotherapy group (*p* = 0.03), while treatment-related mortality was similar in the two groups [44]. Multiple studies have consistently reported the benefit of ASCT by improving the patient’s outcome. The IFM/DFCI 2009 study conducted by Intergroup Francophone Du Myelome and the DETERMINATION study conducted in the United States to evaluate a combination regimen of lenalidomide, bortezomib, and dexamethasone (RVd) versus RVd with ASCT followed by maintenance with lenalidomide in NDMM patients reported higher response rates and improved PFS in ASCT groups (35.0 and 46.2 months, respectively, in RVd arms and 47.3 and 67.5 months, respectively, in RVd + ASCT arms). No OS benefit was observed; however, the studies concluded that MRD was a strong predictor of outcomes associated with longer PSF and OS in IFM/DFCI 2009, and the impact of maintenance therapy with lenalidomide till disease progression was supported by superior PFS in the DETERMINATION study versus IFM/DFCI 2009. The lack of OS benefit of RVd and ASCT in both studies was probably associated with the subsequent treatment options approved over the past years [45,46,47]. For four decades already, ASCT remains the standard treatment for NDMM patients eligible for transplantation [10]. The question of whether the newly approved therapies including CAR T cell therapy can be an alternative to ASCT is still under discussion, and clinical trials to explore this option are ongoing.

**Figure 1:**
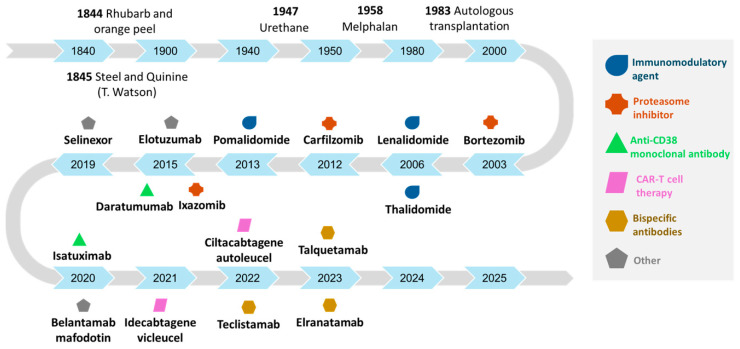
Drugs approved in multiple myeloma as per year of first approval by the FDA.

Tremendous progress has been achieved in multiple myeloma management in the past 25 years with the approval of IMiDs, PIs, and anti-CD38 mAbs, resulting in a major paradigm shift in myeloma treatment (Figure 1).

Numerous combinations of these drugs as doublet, triplet, or quadruplet regimens have been developed for first-line treatment and relapsed or refractory disease. What can be concluded from the huge amount of data generated in many clinical trials is that triplet combinations are superior to doublet combinations, while quadruplet combinations are superior to triplet combinations. The PERSEUS trial compared daratumumab, bortezomib, lenalidomide, and dexamethasone (Dara-VRd) to VRd in transplant-eligible NDMM patients. At 4 years follow-up, the PFS was superior in the Dara-VRd arm than in the VRd arm (84.3% vs. 67.7% *p* < 0.001), and the rate of CR or better in the Dara-VRd arm was higher than in the VRd arm (87.9% vs. 70.1%), while the MRD-negativity status (10^−5^ sensitivity threshold) was 75.2% in the Dara-VRd arm and 47.5% in the VRd arm (*p* < 0.001) [26]. The BENEFIT trial was designed to evaluate the MRD negativity status (at a sensitivity threshold of 10^−5^) at 18 months with the addition of weekly bortezomib to isatuximab (Isa)-Rd (Isa-VRd vs. Isa-Rd groups) in transplant-ineligible NDMM patients. It also reported superior CR or better rates in the Isa-VRd group (58% vs. 33%, *p* < 0.0001), with a higher MRD negativity status in the Isa-VRd group than in the Isa-Rd group (53% vs. 26%, *p* < 0.0001) [48]. The higher MRD negativity rate reported in the PERSEUS trial versus the BENEFIT trial suggests again that ASCT remains the standard treatment in NDMM patients eligible for transplantation. In addition, the IMROZ trial conducted in transplant-ineligible NDMM patients also reported superior efficacy with the quadruplet Isa-VRd combination versus the triplet VRd combination, with an estimated median PFS of 63.2% in the Isa-VRd group and 45.2% in the VRd group (*p* < 0.001) and significantly higher CR or better rates and a MRD-negative status in the Isa-VRd group than in the VRd group (74.7% and 55.5%, respectively, vs. 64.1% and 40.9%, respectively) [27]. Moreover, 26% of patients in the IMROZ trial [27] and 31% of patients in the BENEFIT trial [48] who received the Isa-VRd regimen were ≥75 years old, supporting the feasibility of a quadruplet combination in elderly fit patients.

Despite multiple treatment options and tremendous improvements in patient’s outcome over the past decades and a proportion of patients potentially being cured after 10 years, this disease remains incurable, and almost all patients eventually relapse. Moreover, the duration of remission decreases with each further regimen, and thus, the prognosis of patients with relapse or refractory disease remains poor [49]. An analysis performed in 14 academic institutions in the U.S. showed that the median OS of patients refractory to anti-CD38 monoclonal antibodies was 8.6 months, while for “penta-refractory” patients (defined as having refractoriness to all the five main myeloma treatments, including lenalidomide, pomalidomide, bortezomib, carfilzomib, and either daratumumab or isatuximab), the median OS was only 5.6 months [50]. In the LocoMMotion study conducted in patients with prior exposure to triple-class therapy (PIs, IMiDs, and anti-CD38 mAbs), the overall response rate was 29.8% with a median PFS of 4.6 months and a median OS of 12.4 months with a standard of care treatment regimens including corticosteroids, PIs, IMiDs, alkylating agents, anti-CD38 mAbs, anthracyclines, topoisomerase inhibitors, and other antineoplastic agents. In this context, multiple myeloma remains an unmet need, thus calling for the development of new therapies with a novel mechanism of action [51].

## 5. The Era of T Cell-Directed Therapies in Multiple Myeloma

The increased dysfunction of the innate and adaptive immune system especially in the T cell repertoire such as low T cell count, inverted CD4+/CD8+ ratio, increased levels and functionality of immunosuppressive regulatory T cells (Treg) and proinflammatory Th17 cells, acquired T cell exhaustion or immunosenescence phenotypes, reduced expression of activation markers, and the loss of stem-like/tissue-resident memory (TRM) T cells represent some of the hallmarks of advanced multiple myeloma. This requires the development of additional therapeutic approaches to activate the host’s immune system and to overcome the immunosuppressive tumor microenvironment [52,53,54,55,56,57].

Novel T cell-directed therapies leverage the immune system’s T cells to recognize and attack tumor cells in a non-HLA-restricted manner, combining the antibody-type specificity with effector T cell function [58].

## 6. Chimeric Antigen Receptor (CAR) T Cell Therapies

Autologous CAR T cell therapies emerged as a breakthrough treatment in 2013 [59], followed in 2017 by the approval of the first CAR T cell therapies for patients with some types of aggressive and advanced B-cell acute leukemias and lymphomas. This approach has undergone remarkable evolution in recent years as a result of attempts to improve cell persistence and proliferation, increase cytotoxicity, and ensure a good safety profile [60]. Different engineering strategies have been used, involving the intracellular signaling domain as well as the extracellular domain responsible for antigen recognition. Currently, five generations of CARs have been identified, each of them using progressively evolved strategies to ensure the activation, proliferation, and persistence of CAR T cells in response to a specific antigen. While the first generation of CARs lacked the costimulatory domain, the second generation of CARs incorporated either the CD28 or 4-1BB (also known as CD137 or tumor necrosis factor receptor superfamily member 9—TNFRSF9) costimulatory domains. The third generation of CARs incorporated two or more costimulatory domains to improve efficacy. The fourth and the fifth generations of CARs were developed to produce in addition immunomodulatory molecules in response to antigen stimulation. For example, the latest, fifth-generation CARs are built on the second-generation CARs by incorporating an IL-2 receptor β chain between CD3-ζ and CD28 or 4-1BB, providing a signal transducer and activator of transcription 3 (STAT3) binding site, which activates the Janus kinase (JAK)–STAT signaling pathway. This domain stimulates cell proliferation, prevents terminal differentiation, and shows better persistence [61,62,63].

Both CAR T cell therapies currently approved for the treatment of multiple myeloma, namely idecabtagene vicleucel (Ide-cel) and ciltacabtagene autoleucel (Cilta-cel), are autologous, second-generation CARs directed against B-cell maturation antigen (BCMA). The CARs construct for both Cilta-cel and Ide-cel use 4-1BB as a costimulatory domain, while Cilta-cel has two BCMA-binding domains compared to one binding domain for Ide-cel [64]. Ide-cel was approved in March 2021 based on the phase II KarMMa study results, while Cilta-cel was approved in February 2022 based on the results of the phase Ib/II CARTITUDE-1 study in patients with RRMM after four or more prior lines of therapy including PIs, IMiDs, and anti-CD38 mAbs [7,65].

Both the KarMMa and CARTITUDE-1 studies reported impressive clinical responses in the heavily pretreated population (median of six prior treatment lines; a total of 26% of patients in the KarMMa study and 42% of patients in the CARTITUDE-1 study being penta-drug refractory), with a CR/sCR rate of 33% in the KarMMa study, an 82.5% sCR rate in the CARTITUDE-1 study, and improved PFS and overall survival [64,66,67,68] (Table 2).

The unprecedented results obtained in patients previously exposed to multiple treatment lines supported the development of CAR T cell therapy earlier in the patient’s journey. Based on the results of the phase III CARTITUDE-4 study, Cilta-cel was approved in April 2024 for use in patients with RRMM who had received at least one prior line of treatment and were refractory to lenalidomide. In addition, based on the results of the KarMMa-3 trial, Ide-cel has been approved for use after two or more prior treatment lines including an IMiD, a PI, and an anti-CD38 mAb. Both these studies demonstrated statistically significant and clinically meaningful improvements in PFS and response rates compared to standard care regimens [28,69,70] (Table 2).

The use of CAR T cell therapies in early treatment lines is also supported by the recent changes in the treatment paradigm that utilizes quadruplet regimens at induction and triplet regimens at first or second relapse, leading to triple-class utilization and refractoriness [71]. The attrition rate also increases with each treatment line, and only 8% of non-transplanted patients and 22% of transplanted patients receive a fifth-line therapy, supporting the need to use the most optimal treatment regimens upfront rather than reserving them for later lines of therapy where the clinical benefit may decrease [72]. The fitness of the T cells and the potentially lower tumor burden in early versus late course of the disease also support the better utilization of CAR T cell therapies in earlier treatment lines [73].

The administration of CAR T cell therapy in an elderly population was evaluated in a meta-analysis of 14 prospective clinical trials and observational studies that included 558 patients, of which 146 patients were over 65 years old. The analysis concluded that the response rates and the cytokine release syndrome (CRS) incidence in older adults treated with anti-BCMA CAR T cell therapy were comparable to younger adults but with an increased rate of neurotoxicity in older patients (15% in older adults compared with 6% in younger adults), though statistical significance could not be determined. The results suggest that the administration of CAR T cell therapy is feasible in the older population, and that the chronological age alone should not be an exclusionary criterion for this efficacious therapy [74].

Although the approved CAR T cell therapy demonstrated excellent responses with efficacy noted across different subgroups including in patients with EMD, the presence of EMD has been identified as a risk factor for early progression after CAR T cell therapy [69]. A large multicenter retrospective study conducted in three U.S. academic centers concluded that the incidence of CRS and immune effector cell-associated neurotoxicity syndrome (ICANS) was comparable, and the response rates were also similar between the EMD vs. non-EMD cohorts (ORR 78% vs. 96%, *p* = 0.44) treated in a real-world setting with Ide-cel and Cilta-cel. However, patients with EMD experienced a shorter PFS (6.5 months vs. 9.8 months, *p* = 0.017) and OS (12.9 months vs. 19.4 months, *p* = 0.013) compared to patients without EMD, raising the need for risk-mitigation strategies before and after CAR T cell therapy to prevent disease progression and to improve survival of patients with EMD [75].

The CAR T cell therapies are currently established treatment options, offering remarkable responses in RRMM. However, given the risk of immune-mediated reactions such as CRS and ICANS due to CAR T cell expansion, patients require close monitoring, preferably in specialized centers with multi-disciplinary teams in place.

Although the CRS rates reported in pivotal trials are high, the incidence of CRS grade ≥ 3 is low. Generally, in pivotal clinical trials, the ICANS and other non-ICANS neurotoxicity incidences were less frequent than CRS [28,64,66,67,68,69,70] (Table 2). A retrospective real-world evidence analysis performed by the US Myeloma CAR T Consortium reported comparable safety data while using commercial products. The CRS rate was 81% (of which 7% was CRS grade ≥ 3) in patients treated with Cilta-cel and 82% (of which 3% was CRS grade ≥ 3) in patients treated with Ide-cel in clinical practice.

The neurotoxicity observed in clinical practice with both Cilta-cel and Ide-cel was also comparable with the ones reported in pivotal trials [76,77]. In addition, a small subset of patients treated with anti-BCMA CAR T cell therapy developed other neurological symptoms including Parkinson-like features. An analysis conducted in the USA utilizing the FDA Adverse Events Reports System database reported that out of one hundred seventy-seven patients treated with Ide-cel who reported nervous system disorders, two had features of Parkinsonism, while out of eighty-one patients treated with Cilta-cel who reported nervous system disorders, five had features of Parkinsonism. Further studies and long-term surveillance are needed in order to fully evaluate and characterize the long-term neurotoxicity profile of CAR T cell therapies, including those targeting BCMA [78].

Guidelines and recommendations for diagnosis, grading, evaluation, and management of immune-mediated toxicities are available to support clinical practice. While the management of patients with CRS includes treatments with tocilizumab with or without steroids, ICANS requires prompt intervention with steroids and the best supportive care. In addition, other potential toxicities such non-ICANS neurotoxicity, prolonged cytopenia, B-cell aplasia, hypogammaglobulinemia, infections, and secondary malignancies require regular monitoring and prophylactic measurements and intervention. Moreover, patients exposed to CAR T cell therapies should be monitored for up to 15 years following their infusion as per the regulatory requirement [79,80,81].

### 6.1. Procedures Before and After CAR T Cell Therapy

**Figure 2:**
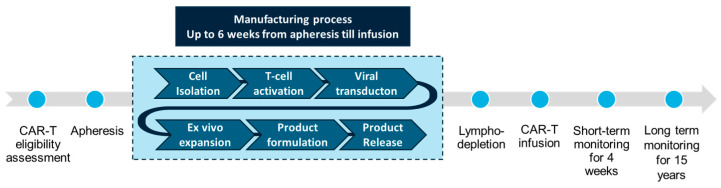
Procedures before, during, and after CAR T cell therapy.

In addition to the challenges posed by the safety profile, the logistic aspects of CAR T cell administration represent another limitation that restricts the widespread use of this therapy. The manufacturing and administration of CAR T cell therapy is a complex process, requiring specialized centers with extensive certification, training, resources, and infrastructure, along with close coordination among healthcare providers across institutions to ensure the process is carried out as smoothly as possible [82] (Figure 2). Once considered eligible for CAR T cell therapy, the patient first undergoes a recommended washout period to minimize the impact of prior therapies on T cell fitness and on CAR T cell manufacturing [83]. Then, apheresis is performed to collect T cells from the patient’s peripheral blood, and the success of CAR T cell therapy substantially depends on the quality of the leukapheresis product. Subsequent CAR T cell manufacturing process and release time range from 3 to 5 weeks on average, depending on the manufacturer’s approved methods and validated processes used for transduction and T cell expansion [84]. The lengthy “vein-to-vein” time along with the limited manufacturing slots and the risk of manufacturing failure are constant logistical challenges which can limit patients access to therapy [85]. During the manufacturing time, bridging therapy might be needed to control the disease, especially in patients with high disease burden and in rapidly progressive diseases. The selection of bridging therapy should be tailored based on patient and disease characteristics, prior treatments, potential toxicities, and future impact on CAR-T cell administration [86]. Prior to CAR-T cell infusion, patients usually receive lymphodepleting chemotherapy with cyclophosphamide and fludarabine. CAR T-administering centers must ensure the availability of at least one dose of tocilizumab (or other suitable alternative option in case of shortage) for use in the event of CRS and access to an additional dose within 8 h of each previous dose. Patients must remain in proximity (within 2 h of travel) of the treatment facility for at least 4 weeks following the infusion of CAR T cells [87,88].

With expanding indications for CAR T cell therapy and improvements in monitoring and managing immune-mediated reactions, administering CAR T cells in the outpatient setting is of high interest to improve access to therapy, reduce financial burden, and increase patients’ convenience. Several small studies have explored CAR T cell administration in an outpatient setting [89], and recently, the American Society for Transplantation and Cellular Therapy published an expert panel article outlining several clinical and logistical factors that needs to be considered. Adequate patient selection, product characteristics such as toxicity profile and time to onset of CRS and ICANS, proximity to the treatment center, access to qualified healthcare personnel for the management of adverse events, education about CAR T cell therapy for staff, patients, and caregivers, and appropriate caregiver support are a few key factors that lead to a successful CAR T cell administration in the outpatient setting [90].

### 6.2. CAR T Cell Therapy Beyond Current Registration in Multiple Myeloma

Despite the logistic complexity and clinical challenges, CAR T cells have demonstrated remarkable results in patients with RRMM and increasingly as an upfront treatment in NDMM (e.g., the CARTITUDE-5 study {NCT04923893} that evaluated the efficacy of VRd induction followed by Cilta-cel versus VRd induction followed by Rd maintenance in patients with NDMM not intended for transplantation [91] and the CARTITUDE-6 study {NCT05257083} that randomized patients with NDMM eligible for transplantation to receive either ASCT or Cilta-cel after frontline induction with DVRd [92], while the KarMMa-4 study {NCT04196491} that evaluated Ide-cel therapy in patients with high-risk NDMM [93]).

In addition, other tumor antigens such G-protein-coupled receptor class C group 5 member D (GPRC5D), signaling lymphocyte activation molecular family 7 (SLAMF7), CD38, CD138, CD229, Fc receptor-homolog 5 (FcRH5), or a proliferation-inducing ligand (APRIL) are being tested as potential CAR T targets, and some of them have progressed from preclinical studies to early phase clinical trials, showing promising initial results [94,95]. GPRC5D expression that is limited to hair follicles and skin made it a promising immunotherapeutic target in multiple myeloma. In a phase I study published by Mailankody S. et al., in 17 heavily pretreated patients, the response rate was 71% with GPRC5D-targeted CAR T cell therapy (MCARH109), while the CRS rate was 88% (grade 1 and 2 in all patients except in one patient who experienced a grade 4 CRS event); a total of 65% of the patients experienced nail changes, and 12% experienced dysgeusia, while two patients had grade 3 cerebellar disorder [95].

Engineering strategies such CAR constructs utilizing natural receptor- or ligand-based designs instead of scFv-based designs, dual-targeted CARs designed either as tandem or bicistronic CARs or “CAR-pools” [96], or armored 4th generation TRUCK CAR T cells incorporating cytokines to increase CAR-T cell infiltration, binding, and proliferation are currently being explored [97].

Moreover, academic strategies are also in place to improve patient access to CAR T cell therapy. For example, in Spain, the CAR T ARI0002h developed at the Hospital Clinic Barcelona-IDIBAPS has been approved by the Spanish authorities as a non-industrially manufactured product for the treatment of patients with relapsed multiple myeloma. (https://www.clinicbarcelona.org/en/news/green-light-for-ari0002h-car-t-developed-by-clinic-idibaps-for-patients-with-multiple-myeloma (accessed on 29 August 2024)

Allogeneic “off-the-shelf” CAR T cells developed from healthy donor T cells could solve some of the current challenges related to the manufacturing of autologous products. Potential graft-versus-host disease (GVHD) and CAR T cell rejection could be theoretical challenges with allogeneic products. Transcription activator-like effector nuclease (TALEN) gene editing has been used to knock out two critical genes, the TCRα subunit constant gene (*TRAC*) and CD52, in order to circumvent CAR T cell rejection and GVHD [98]. In the phase 1 UNIVERSAL study (NCT04093596) conducted to evaluate the safety and efficacy of ALLO-715 BCMA allogenic CAR T cells in patients with RRMM, the CRS rate was 55.8% with only one grade ≥ 3 event, and the neurotoxicity rate was 14% with no grade ≥ 3 events. At the optimal dosing regimen, seventeen out of twenty-four patients (70.8%) had a clinical response, including six patients (25%) with a CR/sCR. The median duration of response was 8.3 months [99].

The future of CAR T cell therapy in myeloma is promising as new CAR constructs under development are aimed to target beyond BCMA, and other potential engineering strategies are being explored to improve the efficacy and safety of the cell products [94].

### 6.3. Bispecific Antibodies (BsAbs)

The T cells that engage bispecific antibodies (BsAbs) are another T cell-directed treatment option for patients with RRMM. The BsAbs simultaneously bind to a tumor antigen BCMA or GPRC5D expressed on myeloma cells and to CD3 on the T cells, resulting in the redirection of T cells to myeloma cells with subsequent T cell activation, leading to tumor cell death [100]. In contrast to autologous CAR T cell therapy, BsAbs are “off-the-shelf” products that do not require apheresis, T cell collection, or individualized manufacturing [101]. The two anti-BCMA BsAbs, teclistamab and elranatamab, and the anti-GPRC5D talquetamab are currently approved for the treatment of patients with RRMM who have received ≥4 lines of therapies (FDA-approved labels), including PIs, IMiDs, and anti-CD38 mAbs, based on the results from pivotal trials [102,103,104] (Table 3).

Immune-mediated reactions are also observed with BsAb treatments, with CRS (mainly grade 1 and 2) being reported in up to 80% of patients (Table 3). Close monitoring, step-up dose, premedication, and prompt intervention with tocilizumab are also required during treatment with BsAbs. Infections are common during treatment, and adequate monitoring and infection prophylaxis are essential measures [110]. In addition, patients treated with talquetamab develop skin-related events, dysgeusia, and nail-related events due to GPRC5D expression in cells that produce hard keratin and are on-target, off-tumor effects of the drug [111].

Bispecific antibodies targeting FcRH5 are currently under development. Cevostamab, a T cell-engaging BsAb that targets FcRH5 on myeloma cells and CD3 on T cells, reported promising results in a phase I dose escalation study [112].

Several combination strategies (e.g., IMiDs, checkpoint inhibitors, and anti-CD38 mAbs) aimed to further improve the response of BsAbs are currently being investigated (e.g., the MajesTEC-2 study [NCT04722146], the MagnetisMM-4 study [NCT05090566], and the MonumenTAL-2 study [NCT05050097]). Moreover, BsAbs are also being evaluated for upfront treatment in NDMM. MajesTEC-7 [NCT05552222] is a phase 3 randomized study that is evaluating Teclistamab +Daratumumab + Lenalidomide in NDMM patients either ineligible or not suitable for ASCT [113], while MagnetisMM-7 [NCT05317416] is a phase 3 randomized study that is evaluating elranatamab versus lenalidomide in NDMM patients who are MRD positive post ASCT [114].

## 7. Conclusions

The efforts made over the past decades to better understand and characterize the complex biology and heterogeneity of multiple myeloma has led to more precise management of the disease and better patient outcomes. The risk stratification tools have evolved to incorporate deep molecular characterization, supporting personalized treatment approaches. With the emerging of immune therapies, the treatment strategy has changed drastically over the recent years.

In the era of quadruplet regimens, CAR T cell therapies and bispecific antibodies have shown unprecedented results in all disease settings (NDMM and RRMM); several clinically relevant questions still remain, such as the future role of ASCT in the emerging treatment landscape, the role of MRD status to inform the treatment strategy in clinical practice, the optimal sequencing strategy in relapsed or refractory setting to maximize the patients’ outcome, the characterization of the long-term safety profile of T cell-engaging therapies including neurotoxicity, and potential second malignancies. Moreover, patients with EMD and plasma cell leukemia are still a high unmet medical need that requires additional strategies to improve the outcome.

Simultaneously, efforts are being made to further investigate the biology and to enhance the risk stratification models of monoclonal gammopathy of undetermined significance (MGUS) and smoldering multiple myeloma (SMM) and to determine the optimal treatment approach especially in high-risk MGUS/SMM in order to influence their natural course of the disease and progression to multiple myeloma.

Racial and ethnic differences are also prevalent in multiple myeloma, and with the ongoing expansion of treatment options, the evaluation of efficacy and safety of the drugs across diverse patient ethnicities is important and needs to be considered in clinical trials.

With the ongoing clinical studies evaluating the T cell engagers as an upfront treatment and in different combinations and early developments exploring CAR T cell therapies with different CAR constructs beyond BCMA, the future of multiple myeloma looks very dynamic and promising and will likely result in the continued improvement of patient’s outcome.

## Figures and Tables

**Table 1:** Risk stratification via ISS in multiple myeloma.

MM Stage	ISS (2005) [14]	R-ISS (2015) [16]	R2-ISS (2022) [17] Points: ISS II = 1; ISS III = 1.5, High LDH = 1; del(17p) = 1; t(4;14) = 1; 1q+ = 0.5
Stage I	Serum β2M < 3.5 mg/L Serum albumin ≥ 3.5 g/dL	ISS stage I Standard-risk cytogenetics Normal LDH level	Low Risk: 0 points
Stage II	Not ISS stage I or III	Not R-ISS stage I or III	Low-intermediate Risk: 0.5–1 points
Stage III	Serum β2M ≥ 5.5 mg/L	ISS stage III High-risk cytogenetics del(17p) and/or t(4;14) and/or t(14;16) or High LDH level	Intermediate-high Risk: 1.5–2.5 points
Stage IV	NA	NA	High Risk: 3–5 points

Abbreviations: ISS, international staging system; LDH, lactate dehydrogenase; MM, multiple myeloma; NA, not applicable.

**Table 2:** Approved CAR T cell therapies in relapsed/refractory multiple myeloma.

Features	KarMMa [66,67] (Ide-Cel)	CARTITUDE-1 [64,68] (Cilta-Cel)	KarMMa-3 [69,70] (Ide-Cel)	CARTITUDE-4 [28] (Cilta-Cel)
Number of patients infused	128	97	225	176
No. of prior treatment lines, median (ranges)	6 (3–16)	6 (3–18)	3 (2–4)	2 (1–3)
ORR (%)	73	97.9	71	84.6
≥CR/sCR (%)	33	82.5	43.7	73.1
MRD negativity (%) (<10^−5^)	26	44.3	22.4	60.6
Median PFS (months)	8.6	34.9	13.8	Not reached (75.9% at 12 months)
Median OS (months)	24.8	Not reached (62.9% survival at 36 months)	41.4	Not reached (84.1% at 12 months)
CRS, any grade (%)	84	95	88	76
CRS grade ≥ 3 (%)	5	4	5	1.1
ICANS any grade (%)	18	17	15	4.5
ICANS grade ≥ 3 (%)	3	2	3	0
Non-ICANS and delayed neurotoxicity any grade (%)	0	12.4	0	17

Abbreviations: CR, complete response; CRS, cytokine release syndrome; ICANS, immune effector cell-associated neurotoxicity syndrome; MRD, minimum residual disease; ORR, overall response rate; OS, overall survival; PFS, progression-free survival.

**Table 3:** Approved bispecific antibodies in relapsed/refractory multiple myeloma.

Features	Elranatamab [105,106]	Talquetamab [107,108]	Teclistamab [29,109]
Target antigen	BCMA/CD3	GPRC5D/CD3	BCMA/CD3
Indication	RRMM who have received at least four prior lines of therapy, including a PI, an IMiDs, and an anti-CD38 mAb.		
Pivotal trial/Phase	MagnetisMM-3 Phase II	MonumenTAL-1 Phase II	MajesTEC-1 Phase II
No. of patients	123	130 (subcutaneous cohort)	165
Median no. of prior treatment lines (ranges)	5 (2–22)	6 (2–17)	5 (2–14)
ORR (%)	61	74 ^a^	63
≥CR rate (%)	37.4	33.6 ^a^	46.1
Median PFS (months)	17.2	7.5 ^a^	11.4
Median OS (months)	24.6	Not reached (76.4% 12-month OS ^a^)	22.2
CRS (%)	57.7	80 ^b^	72.1
Neurologic events (%)	4.9	10 ^b^	14.5
Infection (%)	70.7	47 ^b^	79
Skin related events (%)	-	70 ^b^	-
Dysgeusia (%)	-	63 ^b^	-
Nail-related event (%)	-	57 ^b^	-

^a^ Data from the subcutaneous treatment cohort 405 mcg QW; ^b^ Data from any subcutaneous treatment cohort with the highest incidence reported. Abbreviations: BCMA, B-cell maturation antigen; GPRC5D, G-protein-coupled receptor class C group 5 member D; PI, proteasome inhibitor; mAb, monoclonal antibody; IMiDs, immunomodulatory drugs; ORR, overall response rate; CR, complete response; OS, overall survival; PFS, progression-free survival; CRS, cytokine release syndrome.

## Data Availability

All data and references mentioned in this manuscript are from publicly available sources. Data sharing is not applicable for this article as no datasets were generated or analyzed during the current study.
